# An *Arabidopsis* introgression zone studied at high spatio-temporal resolution: interglacial and multiple genetic contact exemplified using whole nuclear and plastid genomes

**DOI:** 10.1186/s12864-017-4220-6

**Published:** 2017-10-23

**Authors:** Nora Hohmann, Marcus A. Koch

**Affiliations:** 1 Center for Organismal Studies (COS) Heidelberg/Botanic Garden and Herbarium Heidelberg (HEID), University of Heidelberg, Im Neuenheimer Feld 345, D-69120 Heidelberg, Germany; 2 Present address: Department of Environmental Sciences, Botany, University of Basel, Hebelstrasse 1, CH-4056 Basel, Switzerland

**Keywords:** *Arabidopsis*, Adaptive introgression, Phylogenomics, Suture zone

## Abstract

**Background:**

Gene flow between species, across ploidal levels, and even between evolutionary lineages is a common phenomenon in the genus *Arabidopsis*. However, apart from two genetically fully stabilized allotetraploid species that have been investigated in detail, the extent and temporal dynamics of hybridization are not well understood. An introgression zone, with tetraploid *A. arenosa* introgressing into *A. lyrata* subsp. *petraea* in the Eastern Austrian Forealps and subsequent expansion towards pannonical lowlands, was described previously based on morphological observations as well as molecular data using microsatellite and plastid DNA markers. Here we investigate the spatio-temporal context of this suture zone, making use of the potential of next-generation sequencing and whole-genome data. By utilizing a combination of nuclear and plastid genomic data, the extent, direction and temporal dynamics of gene flow are elucidated in detail and Late Pleistocene evolutionary processes are resolved.

**Results:**

Analysis of nuclear genomic data significantly recognizes the clinal structure of the introgression zone, but also reveals that hybridization and introgression is more common and substantial than previously thought. Also tetraploid *A. lyrata* and *A. arenosa* subsp. *borbasii* from outside the previously defined suture zone show genomic signals of past introgression. *A. lyrata* is shown to serve usually as the maternal parent in these hybridizations, but one exception is identified from plastome-based phylogenetic reconstruction. Using plastid phylogenomics with secondary time calibration, the origin of *A. lyrata* and *A. arenosa* lineages is pre-dating the last three glaciation complexes (approx. 550,000 years ago). Hybridization and introgression followed during the last two glacial-interglacial periods (since approx. 300,000 years ago) with later secondary contact at the northern and southern border of the introgression zone during the Holocene.

**Conclusions:**

Footprints of adaptive introgression in the Northeastern Forealps are older than expected and predate the Last Glaciation Maximum. This correlates well with high genetic diversity found within areas that served as refuge area multiple times. Our data also provide some first hints that early introgressed and presumably preadapted populations account for successful and rapid postglacial re-colonization and range expansion.

**Electronic supplementary material:**

The online version of this article (doi: 10.1186/s12864-017-4220-6) contains supplementary material, which is available to authorized users.

## Background

Hybridization is an important driver for speciation [1], particularly in plants [2]. Frequently, hybridization is associated with polyploidization (allopolyploidization); and polyploid speciation (neopolyploidization; see [3]) events have been estimated to make up 24% of all speciation events in present-day angiosperms, with almost half of them allopolyploidizations [4]. This high frequency is thought to be linked to the advantages of (allo-) polyploids compared to their diploid ancestors. Through the loss of genetic constraints on duplicated alleles, new gene functions and gene families can evolve [5]. Many allopolyploid species are able to colonize new ecological niches, and these are not necessarily restricted to niches intermediate of their ancestors [6].

A special case of hybridization is introgression, where as a consequence of back-crossing with one of the parental species the genomic contribution of one species is increasingly larger than the other parent’s. This can result in clinal genetic and ecological differentiation [7–9]. With the genomic tools now available, introgression has become a popular study area, particularly where genes of adaptive value introgress preferentially. This so called ‘adaptive introgression’ has been described from a number of species in both animals [10] (e.g. *Heliconious* butterflies [11]; sticklebacks [12]) and plants (e.g. sunflower [13], *Arabidopsis* [14]; *Silene* [15]; *Populus* [16]).

The model genus *Arabidopsis* provides great opportunities to study hybridization and introgression, as several cases of hybridization have been documented between *Arabidopsis* species and even between major evolutionary lineages. Two stabilized allopolyploid species, *A. suecica* [17–20] and *A. kamchatica* [21–24] have been described. Also gene flow between different *Arabidopsis* species is more common [25] than previously assumed, and is found even across ploidal levels [26]. Our introgression study system was first characterized by Schmickl and Koch [7], who reported a suture zone stretching from the eastern Austrian Forealps in the south to the Danube valley in the Wachau region and further to the north-eastern border of Austria. In this area *A. arenosa* (hereafter called ‘Arenosa’) introgressed into *A. lyrata* (hereafter called ‘Lyrata’). This region is also a major center of genetic diversity for both Lyrata and Arenosa [27]. A first indication of the hybridization was given by the morphological intermediacy of many Lyrata populations in the area, and subsequent analyses showed that the respective populations are indeed exclusively tetraploid [7].

Taxonomically, the Arenosa populations in Austria can be assigned to the tetraploid mountain taxon *A. arenosa* subsp. *borbasii*, with only few populations at rural sites (railway tracks, e.g.) belonging to the tetraploid lowland taxon *A. arenosa* subsp. *arenosa* [28], both of which most likely originated through autopolyploidization [29–31]. In contrast, Lyrata populations in the area belong to the European subspecies *A. lyrata* subsp. *petraea*, which is generally diploid in Central Europe with few local exceptions [24, 32]. However, the introgressed tetraploid Lyrata populations represent fully stabilized polyploids. Evidence from the maternally inherited plastid genome suggests that gene flow is unidirectional and Lyrata acts as the maternal parent, while Arenosa is the pollen donor [7, 33]. Asymmetrical gene flow is frequently found among natural *Arabidopsis* hybrids and is often favoring Lyrata as maternal parent [32]. In the offspring of artificial crosses between diploid Lyrata and diploid representatives of the Arenosa group from the Carpathian Mountains, asymmetrical fitness has been detected and has been ascribed to cytonuclear incompatibilities [33]. However, the fitness of Arenosa maternal hybrids was found to be higher in these crosses, while in our study area Lyrata type plastids are found indicating higher fitness in Lyrata maternal hybrids [7].

It has been hypothesized that the first hybrids between Lyrata and Arenosa in the Wachau occurred within the Late Pleistocene, and subsequent secondary contact at the northern edge of the introgression zone was thought to be postglacial [7]. The allotetraploid species in *Arabidopsis*, *A. suecica* and *A. kamchatica*, also both likely originated during the Late Pleistocene [17, 21]; and *A. suecica* is most likely of postglacial origin less than 20 kya (thousand years ago) [19]. On a larger geographic scale gene flow between the two study species and even across ploidal levels has been described extensively before [7, 26], however data available to date are not appropriate to estimate temporal evolutionary scenarios of hybridization and gene flow due to limited marker resolution and evolutionary models to be applied (e.g., in case of microsatellites). Additionally, extensive gene flow between all lineages of *Arabidopsis*, including *A. thaliana* [25], suggests that reproductive isolation in *Arabidopsis* is rather caused by geographic isolation than genetic incompatibilities and that gene flow may have occurred even long after lineage diversification [25].

Present-day species distribution patterns and vegetation composition in Central Europe is heavily influenced by glaciation and deglaciation cycles during the Pleistocene and in particular by the last three major glaciation/deglaciation cycles with three glaciations or glaciation complexes [named Elster (480–410 kya), Saalian (380–130 kya), Weichselian (115–11.7 kya) in Northern Europe; or Mindel, Riss, Würm in the European Alps, respectively] [34]. Large parts of the Alps were covered with ice and snow during these cold periods, and plants had to retreat to refugia for periglacial survival, from where they recolonized suitable habitats in warming interglacial periods. These refugia were identified mostly at the southern, but also the eastern border of the European Alps [35]. Maximum extent of the ice sheet in the Austrian Alps during the last two glaciation complexes were reached about 20 kya in the Würm and 135 kya in the Riss glaciation [36]. It has been hypothesized that some populations of Lyrata survived the last glacial maximum (LGM, Würm glaciation) north of the Alps [37, 38], and large effective population sizes for the population in Plech, Germany as well as for the ancestral population of Lyrata have been estimated [39, 40]. The diploid Lyrata populations in the Eastern Austrian Forealps that might have served as the maternal parent for introgressed tetraploid Lyrata were also hypothesized to have survived the LGM in the cryptic refugia dominated by open rock habitats and cold-adapted vegetation they still inhabit today [7, 41].

The *Arabidopsis* introgression zone in the Wachau is of particular interest because of its potential to study adaptation to substrate type, increased temperature and drought stress. Diploid Lyrata are usually restricted to limestone and dolomite, whereas tetraploid Lyrata are found on siliceous bedrock, suggesting the acquisition of genes adapted to this bedrock type through introgressive hybridization with Arenosa [7].

The genetic and genomic tools available for *A. thaliana* are easily applicable in the remainder of the genus, thus making *Arabidopsis* an ideal model system for comparative evolutionary biology [42, 43]. Next-generation sequencing (NGS) techniques and high quality genomes sequences from two species of the genus, *A. thaliana* (TAIR: https://www.arabidopsis.org) and *A. lyrata* [44], allow for open questions from previous research now to be investigated in more detail.

With this study, we aim to first test the idea of a unidirectional introgression zone using genome-wide DNA analysis, we further aim to quantify the genetic levels of introgression along the presumed south-north gradient, and finally we elaborate on a spatio-temporal setting for this complex scenario of repetitive Pleistocene genetic isolation and subsequent gene flow.

## Results

We analyzed whole-genome re-sequencing data of Arenosa and Lyrata from an introgression zone in northeastern Austria [7]. We included all closely related taxa from adjacent areas to set our findings into a broader evolutionary context. All of these related taxa belong to the Arenosa group and were sampled in the Carpathian Mountains, and for simplicity those will be addressed here as ‘Carpathian Arenosa’.

### Spatial distribution of genetic variation and genetic admixture

**Fig. 1:**
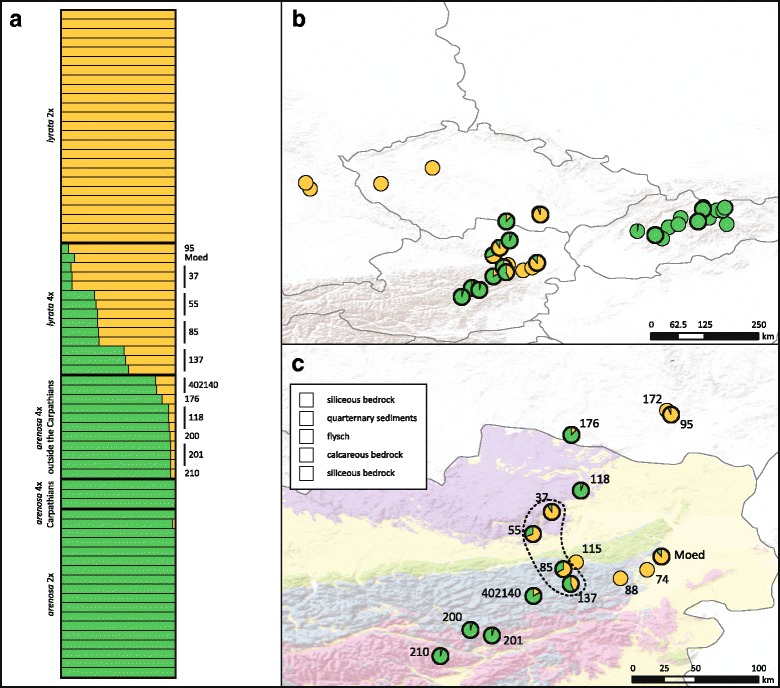
Genetic clustering analysis from STRUCTURE [45]. Results for mean values over 10 independent runs at *K* = 2 are shown. Runs are based on 10 randomly sampled subsets of the complete dataset of 15,454 genes, using a fraction of 0.05 of synonymous sites per subset. **a** bar plot showing the genetic assignment of each sample, sorted by taxon and population. **b** Mean values over all individuals as pie charts in each population in the complete sampling area. **c** Mean values over all individuals in each population in enlarged suture zone in northeastern Austria and adjacent regions. The Wachau region is indicated with a dashed line. Tetraploid populations are marked by black circles around the pie charts. Bedrock type was categorized based on the geological map of Austria (KM500 Austria from http://www.geologie.ac.at) under the Open Data licence Creative Commons 3.0 Österreich (CC BY 3.0 AT)

Genetic clustering was used to detect population structure and affinity to genetic clusters. The STRUCTURE analysis [45] based on nuclear genome-wide SNP calling characterizes the introgression zone. The most likely number of genetic clusters [46] was estimated to K = 2, and similarity among runs with 10 independent, randomly chosen subsets of synonymous SNPs was very high (> 0.97). The two detected genetic clusters generally represent the Arenosa and Lyrata gene pools (Fig. 1). While Carpathian diploid/tetraploid Arenosa and diploid Lyrata show no traces of admixture, tetraploid Arenosa from Austria as well as tetraploid Lyrata were partially assigned to one or the other gene pool. Only two samples of Carpathian Arenosa had a partial, although minute, genetic assignment to the Lyrata cluster (0.2% in SK08-h, *A. petrogena* subsp. *exoleta* and 2% in 915,139-g, *A. petrogena* subsp. *petrogena*). Within tetraploid Lyrata we detected a gradient of introgression from the eastern Austrian Forealps northwards towards the Danube valley in the Wachau region (Fig. 1 b, c), as had been shown previously using a small set of nuclear microsatellites [7]. The mean proportion of Arenosa genome in tetraploid Lyrata was highest in the south of this Wachau suture zone, with a population mean of 57% for pop. 137, and lowest in the northern population with a mean of 9% for pop. 37. Two tetraploid Lyrata populations from outside the introgression zone were also investigated, and both of them also showed partial assignment to the Arenosa cluster: Among them, pop. Moed exhibits an even higher proportion of Arenosa genome (12%) than the northernmost population from the introgression zone. With a genetic assignment of 7% to the Arenosa cluster, pop. 95 (Czech Republic) had an even lower affinity to the Lyrata cluster.

All (tetraploid) Arenosa from the study area also showed signatures of admixture with Lyrata. The southernmost populations carry the smallest amount of Lyrata in their genomes, with only 4%, while the population adjacent to the southern border of the introgression zone showed a much larger proportion of Lyrata genome with 17% (pop. 402,140), thereby also demonstrating higher admixture than the northernmost population of Lyrata in the introgression zone. However, the population adjacent to the introgression zone in the north had a much lower amount of admixture (pop. 118, with 6%). Pop. One hundred seventy six from Czech Republic, well outside the reported introgression zone, also showed a relatively high amount of admixture (12%), as did the highly endemic and high alpine taxon *A. arenosa* subsp. *arenosa* var. *intermedia* (pop. 210, 4%).

Results from phylogenetic network reconstruction using SplitsTree [47] (Additional file 1) were consistent with genetic assignment in STRUCTURE. Both Arenosa and Lyrata were recognized as distinct groups, with diploids at the respective tips of the network and all tetraploids in between. The network also recognized the intermediate position of both the tetraploid introgressed Lyrata and tetraploid Arenosa. Their position followed the level of admixture suggested by STRUCTURE analysis, with pops. 37, 95 and Moed close to diploid Lyrata, and more admixed populations towards the Arenosa group, and with pop. One hundred thirty seven from the southernmost border of the suture zone closest to Arenosa. Also within tetraploid Arenosa, the position towards the Lyrata group depends on the level of admixture. Pops. 402,140 and 176, that showed the highest degree of admixture in STRUCTURE analysis, were closest to each other and closest to the Lyrata group. All other samples from this taxon, as well as endemic high alpine *A. arenosa* subsp. *arenosa* var. *intermedia*, clustered together (Additional file 1).

### Polymorphisms are shared within and between species, cytotypes and populations

**Fig. 2:**
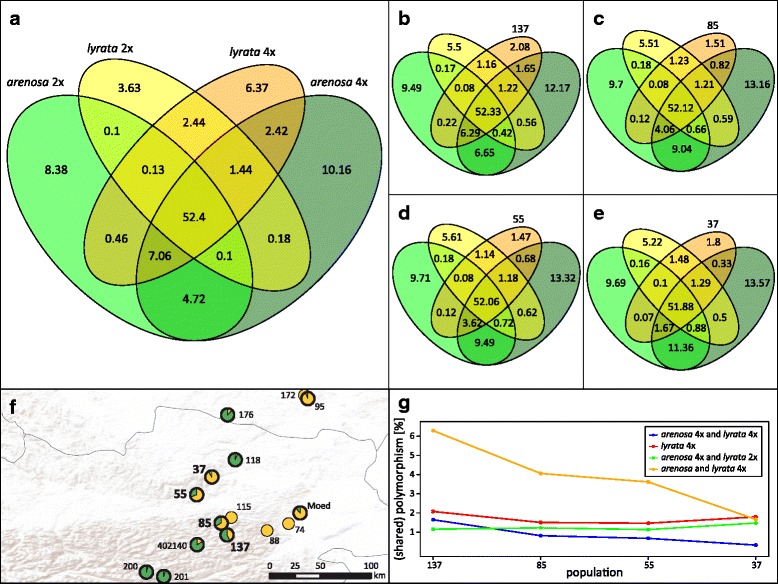
Percentage of shared polymorphism between Lyrata and Arenosa. Only biallelic SNPs were considered, and loci with missing data were ignored. Four groups were evaluated in each analysis: Diploid Arenosa, tetraploid Arenosa, diploid Lyrata and tetraploid Lyrata (A), or the respective population from the introgression zone from south to north only (B-E). **a**: all tetraploid Lyrata were included, **b**: only population 137, **c**: only population 85, **d**: only population 55, **e**: only population 37. **f**: map highlighting the populations in the introgression zone (bold). **g**: Trends across the introgression zone (south to north) for selected examples are shown and were taken from diagrams B to E. The map was created under the Open Data licence Creative Commons 3.0 Österreich (CC BY 3.0 AT)

Considering a scenario of an introgression zone, we expect the introgressed populations to share their polymorphism with either of the parental species. We also expect an increasing amount of polymorphism shared with one of the parental species along a clinal gradient following the suture zone from the South to the North; while the amount of polymorphism shared with the other parental source is decreasing. We assessed shared polymorphism between groups by counting the number of shared SNPs between the investigated groups, namely diploid and tetraploid Lyrata and Arenosa. The results are displayed in Venn diagrams, considering all samples (Fig. 2a) as well as tetraploid Lyrata populations along the introgression zone separately (Fig. 2b-e).

In the dataset comprising all populations of tetraploid Lyrata from the area of interest (pops. 37, 55, 85 and 137), a total number of 2,505,371 biallelic SNPs or 5,010,742 alleles was analyzed. The highest total number of alleles was found within tetraploid Arenosa (78.72%), followed by diploid Arenosa (73.35%), tetraploid Lyrata (72.72%) and finally diploid Lyrata (60.42%). With approximately 52%, more than half of the alleles were shared between all four groups. The amount of private alleles was highest in tetraploid Arenosa (10.16%), followed by diploid Arenosa (8.38%). A high amount of private alleles was also detected in tetraploid Lyrata (6.37%), while diploid Lyrata had the lowest amount of private alleles (3.63%). In contrast, when considering only one of the tetraploid Lyrata populations, the amount of private alleles in tetraploid Lyrata was lower (1.47% - 2.08%).

As expected, the amount of polymorphism shared between tetraploid Arenosa and tetraploid Lyrata was decreasing (from 61.49 to 55.17%; Fig. 2b-e) following the introgression zone from south to north. However, the amount of polymorphism shared between diploid and tetraploid Lyrata did not differ significantly (varying between 54.46 and 54.79%, Fig. 2b-e). A more detailed perspective is provided with Fig. 2g, and the most severe decrease along the cline from south to north is found with uniquely shared polymorphisms between Arenosa and tetraploid Lyrata (from 6.29 to 1.67%).

### Phylogenetic reconstruction based on the plastid genome provides the maternal perspective

**Fig. 3:**
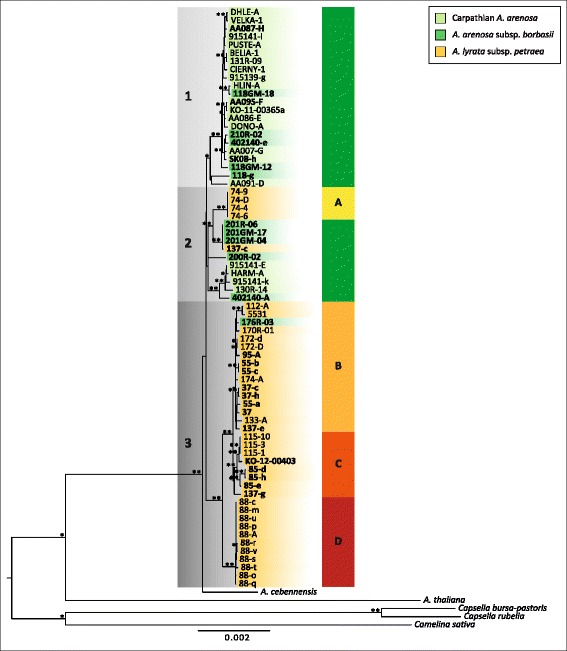
Maximum likelihood phylogenetic reconstruction of complete plastid genome sequences from Lyrata and Arenosa in Central Europe. Bootstrap values from 1000 bootstrap replicates are indicated (** for bootstrap support of 100% and * for support of 95–99%). *Capsella bursa-pastoris*, *Capsella rubella* and *Camelina sativa* were set as outgroup. Taxa are indicated with color codes at sample names, tetraploids are indicated in bold. Four clades of Lyrata are categorized with a separate color code: yellow, orange and red bars and marked **a, b, c/d**, respectively

To follow the maternal origin of tetraploid Lyrata, we reconstructed a phylogenetic tree based on the complete sequence of the maternally inherited plastid genome using maximum likelihood (ML). *Capsella bursa-pastoris*, *Capsella rubella* and *Camelina sativa* were used as outgroup with sister relationship to *Arabidopsis* [48–50], and from within the genus *Arabidopsis A. thaliana* and *A. cebennensis* were included as outgroup species to Lyrata and Arenosa [25]. Bootstrap values were generally high and above 95%, however the backbone of Lyrata and Arenosa clades had only low support (Fig. 3).

The occurrence of polyploids (indicated in bold) is not restricted to one single clade, but is a recurring pattern across the entire tree (Fig. 3). This applies to both species groups, but may be more astonishing in Lyrata, as this group represents only one taxon in Europe and not several different taxa with distinct ecological niches such as Austrian and Carpathian Arenosa. The two species groups fall into three major clades (indicated in grey in Fig. 3), with one clade shared between them (clade 2). Moreover, also tetraploid Lyrata are found in two major clades (clades 2 and 3), and can furthermore be split into four deeply divergent haplotype groups (A-D in Fig. 3).

**Fig. 4:**
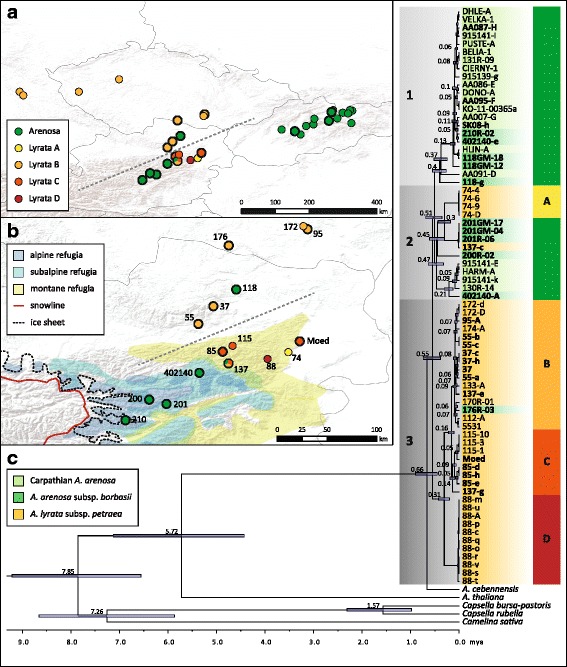
Divergence time estimates based on complete plastid genome sequences from Lyrata and Arenosa in Central Europe. **a**: Map showing plastome clades (defined for Lyrata) and Arenosa plastid types from phylogenetic reconstruction. Color codes correspond to plastome clades from Figs. 3, 4c. Dashed grey line indicates the separation of northern and southern plastome types. **b**: Detailed zoom of map shown in A with information on snowline and ice sheet extensions during the Last Glacial Maximum (20 kya), and refuge area for plant species indicated according to different vegetation zones [35]. Dashed grey line indicates the major biogeographical separation of northern and southern plastome types. The map was created under the Open Data licence Creative Commons 3.0 Österreich (CC BY 3.0 AT). **c**: Combined BEAST results from two independent MCMC runs of 1*10^8^ generations each. Secondary calibration was performed using the results from Hohmann et al. (2015) [3] for the split of the outgroup genera, *Capsella* and *Camelina*, as well as for the *Arabidopsis* crown age and root height. Divergence time estimates are shown with their 95% HPD intervals, only node ages ≥0.05 mya are given. Taxa are indicated with color codes at sample names, tetraploids in bold. Four clades of Lyrata are indicated with yellow, orange and red bars (**a**, **b**, **c**, and **d**)

One individual from the southernmost population, 137-c, has a plastid type closely related to Arenosa pop. 201. These two populations are also geographically close, indicating recent and ongoing gene flow at the southern edge of the introgression zone. Maternal gene flow in the other direction is evident north of the introgression zone, where Arenosa from the southern Czech Republic (pop. 176) carries a Lyrata plastid type B. Also diploid populations of Lyrata can be found in two major clades, with all samples from pop. Seventy four found exclusively in clade 2. Generally, diploid Lyrata investigated here can be divided into four clades (Fig. 3). Three different populations were sampled from Lower Austria, and several individuals from Germany and the Czech Republic. While geographically close groups are not necessarily closely related, individuals within the groups form well supported monophyletic clades with no exceptions. In contrast, no such pattern could be detected in Arenosa, where neither populations nor taxa group together. Apart from the aforementioned 137-c with an Arenosa plastid, tetraploid Lyrata contains plastid types closely related to two of the four groups found in diploid Lyrata. The two populations in the northern part of the introgression zone (pops. 37 and 55) as well as the sample from Czech Republic (pop. 95) are in the clade of Czech and German diploids, and the more southern populations group with Austrian pop. 115 (with the exception of pop. 137, which has plastid types from both groups), thus showing a clear separation into northern and southern clade (indicated by the dashed line in Fig. 4b).

### Divergence time estimations indicate late Pleistocene differentiation

Divergence time estimation was based on the complete plastid genome and conducted in BEAST [51]. The topology of the resulting tree (Fig. 4) is consistent with the results from ML phylogenetic reconstruction, with differences only at splits with low bootstrap support in ML analysis. The crown age of outcrossing *Arabidopsis*, represented here by the split between *A. cebennensis* and the clade of Arenosa and Lyrata, was estimated to 0.66 million years ago (mya), and therefore is predating the last three glaciations [Mindel, Riss, Würm; corresponding to Elster (480–410 kya), Saalian (380–130 kya), Weichselian (115–11.7 kya) in Northern Europe]. The onset of lineage diversification follows shortly thereafter, at 0.55 mya, and within 100 ky the three major plastid lineages split (Fig. 4). Few taxonomically or geographically meaningful deep splits of about 300 kya exist; but most splits are more recent (<140 kya) and most likely peri- and postglacial. Divergence between the four clades in Lyrata is deep, with pop. 74 (clade A) having split from the other 0.55 mya (million years ago) before the Mindel glaciation. However, here chloroplast capture of an Arenosa-plastid type or incomplete lineage sorting might have occurred to explain this finding. Pop. 88 (clade D) has a split time placed in the Early Riss glacial period 0.31 mya. The two clades consistently containing tetraploids (clades B and C) split 0.16 mya during Late Riss glaciation, but before the maximum extent of the ice sheets 135 kya [36]. The deepest split within the group north of the Alps (clade B) is only 0.09 mya, while the crown age of the southern group (clade C) is 0.14 mya, also set during the cold period of Riss glaciation, shortly after divergence from clade B. Very recent divergence between plastid genomes found in Arenosa and Lyrata can be observed in two cases. The split of tetraploid Lyrata sample 137-c from all individuals of pop. Two hundred One from Arenosa is younger than 50,000 years. And the split of the Czech Arenosa sample 176R-03 from its next relative, a plastid type found in diploid Lyrata from Germany is of about 60,000 years of age. Both of these cases are indicative of recent (possibly postglacial) gene flow and plastid capture between the Arenosa and Lyrata lineage. It is worth to mention that also the plastome split between *Capsella rubella* and *Capsella bursa-pastoris* (1.57 mya, 95% confidence interval 0.98–2.31 mya) correlates well with a species split-time estimates (minimum age) of about 1 mya based on whole nuclear genome data and using coalescent models [52].

## Discussion

### Genetic clustering indicates an enlarged suture zone

Genetic cluster analysis significantly shows the introgression of Arenosa into tetraploid Lyrata in our study area (Fig. 1). The amount of genetic contribution of Arenosa is highest in the Eastern Austrian Forealps in the south of the suture zone described earlier from the lowlands [7] and is represented by pop. 137. This is also indicated by SplitsTree analysis, where this population is closest to the Arenosa samples (Additional file 1). Following the sampled populations northwards, the amount of genomic contribution of Arenosa is decreasing, and SplitsTree places the northernmost pop. Thirty seven closest to diploid Lyrata. This is in line with patterns previously reported and described using few microsatellite markers [7]. However, STRUCTURE results show that the extent of introgression is much larger than previously known and expected. Gene flow between Arenosa and Lyrata is possible on the tetraploid level, while none are found in diploids [26, 53]. A congruent result was found here. In STRUCTURE analysis, diploids showed no signs of admixture, with the exception of one Carpathian Arenosa; however, the amount of Lyrata genome in this individual is very low with only 2%. In Lyrata, two tetraploids from outside the introgression zone were investigated, and both of them show traces of the Arenosa gene pool. The population close to Vienna (pop. Moed) was known to be tetraploid before, and had been investigated in several studies [26, 54]. The other population in the Czech Republic however was never studied in detail, and was shown recently to be tetraploid [28]. The amount of Arenosa genome in both these populations is surprisingly high since it was assumed that these populations are typical *A. lyrata* (e.g. based on morphology, [7]), and with 12%, the population from Mödling even has a higher amount than that found in the northernmost population of the introgression zone. Interestingly, the morphological intermediacy between Arenosa and Lyrata that has been reported for the introgression zone [7] cannot be found in Mödling, and the ecology of the population, growing on rocky calcareous outcrops and old limestone walls is very different from that of the introgression zone and is closest to diploid Lyrata; therefore and because of similar ecological niches of diploid Lyrata and tetraploid populations from Mödling an autotetraploid origin had been assumed previously. However, both STRUCTURE results and SplitsTree network reconstruction strongly suggest gene flow from Arenosa into this population as well, thus indicating an enlarged suture zone (including regions around pops. 95 (Czech Republic) and Mödling) and providing first evidence for a more complex spatio-temporal evolutionary scenario.

### Divergence time estimates indicates inter- and periglacial diversification

**Fig. 5:**
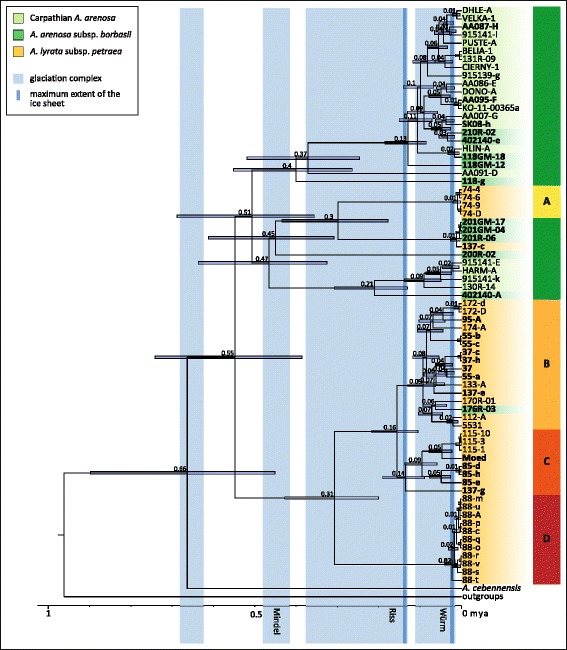
Divergence time estimates based on complete plastid genome sequences from Lyrata and Arenosa in Central Europe. Detail from Fig. 4 with focus on the last ~700 kya. Divergence time estimates are shown with their 95% HPD intervals. Taxa are indicated with color codes at sample names, tetraploids in bold. Four clades of Lyrata are indicated with yellow, orange and red bars (**a**, **b**, **c**, and **d**). Glaciation complexes are indicated in lighter blue, maximum extent of the ice sheets during Würm and Riss glaciation in dark blue lines

Some geographically close populations of diploid Lyrata in the Eastern Austrian Forealps show deep divergence in plastid genome phylogenetic reconstruction. The two south-easternmost populations, pops. Eighty eight and 74, have plastid types divergent from all other Lyrata plastids, and divergence time estimates of 0.31 mya and 0.55 mya, respectively. SplitsTree network analysis based on the nuclear genome data also places these two populations the furthest from the introgression zone, although data from the nuclear genome place them closely together (Additional file 1). However, the occurrence of diploid Lyrata in this region is often restricted to cryptic refugia with open rocks and cold-adapted relic vegetation types, and survival during the last glaciation cycles in these refuge areas has been previously hypothesized [7]. The isolated positions of pops. 88 and 74 in the chloroplast phylogenetic reconstruction as well as in SplitsTree network confirm this hypothesis, and coincides with potential refuge areas for mountain plants on calcareous bedrock in the area in question with sufficient large effective population sizes to maintain high genetic diversity [55] (Figs. 1, 4). Split times among plastid genomes and its correlation with the last four glaciations and respective maximum extent of the ice sheet (if known) is shown with Fig. 5 (detailed blow-up of Fig. 4). It is remarkable that the vast majority of splits in the plastome phylogeny is placed into glaciation periods maybe indicating populations of related species forced into ‘melting suture zones’. The crown group age of tetraploid Lyrata is 0.16 mya (with the exception of sample 137c that carries an Arenosa type plastid) (Figs. 1, 5). This coincides with the ending of the Riss glacial or beginning of the last interglacial period, indicating a first occurrence of tetraploid Lyrata around this time. Ice extent of the glaciers in Eastern Austria at that time reached far into the Foreland [36], and we can hypothesize that species were forced into genetic contact at lower elevations. Signatures of past diversification and contact of gene pools are also evident from the distribution patterns of plastid genetic diversity and are highest in the montane refuge areas of the northeastern Alps (Fig. 4; south of the indicated dashed line). This montane refuge consists of different bedrock types, often changing over short distances and thereby providing the chance for limestone adapted Lyrata and siliceous bedrock adapted Arenosa to come into close genetic contact.

The majority of splits in the plastome tree (Fig. 5) is found during the Würm glaciation indicating an increased net diversification rate. Presumably, effective population sizes were large and allowed to maintain significant proportions of the genetic variation. This has been described earlier for Lyrata populations from northern Bavaria in Germany (exemplified here with pops. 112 and 133) with a split time from Bohemian populations calculated with 39,000 generations (minimum of 78,000 years) and also placed completely into Riss glaciation with effective population sizes of approximately 175,000 individuals [40, 56] estimated an origin of autotetraploid Arenosa populations in the Eastern Alps also to 11,000–30,000 generations ago. Divergence between *A. halleri* and *A. lyrata*, representing the timing of rapid diversification of the major lineages within *Arabidopsis*, was estimated to have occurred 337,000 years ago [57]. Such studies give valuable insights into coalescent-based estimates relevant for the introgression zone, as the establishment of hybrids must postdate the origin of their diploid ancestors. However, compared to the plastid perspective, these estimates are younger (see also Table 1 for a comparison of divergence time estimates in *Arabidopsis*). This could be due to the non-recombining nature of plastid genomes that might lead to overestimated divergence times, in particular when the number of samples is low and coalescent analyses could underestimate divergence in the presence of gene flow, which was reported in high amounts in *Arabidopsis* [25]. Considering the limitations from both approaches however, the first establishment of tetraploid (and introgressed) Lyrata is still likely to have occurred either during the last alpine interglacial period (Riss-Würm interglacial: around 120 kya) or maybe during or around the maximum glaciation of the Riss glaciation (135 kya) with glaciers reaching into the Forelands and thereby also forcing plant populations of related species into ‘melting suture zones’. Two cases of secondary contact with intrapopulational plastid type variation are apparent from phylogenetic reconstruction and BEAST analysis at the extended borders of the introgression zone: (1) tetraploid Arenosa pop. 176R-03 from southern Czech Republic carries a plastid type closely related to diploid Lyrata from Germany (Lyrata type B), and (2) tetraploid Lyrata sample 137-c from the southernmost population of the introgression zone carries a putative Arenosa plastid type, while other representatives of this population carry a typical Lyrata plastid type. This suggests that sample 137-c might have acquired a plastid from Arenosa through chloroplast capture. Both of these instances are relatively recent (peri- or postglacial), and indicate that gene flow between major evolutionary lineages in *Arabidopsis* is an ongoing process rather than a concluded event (or series of events) from the past.

**Table 1:** Divergence time estimates in *Arabidopsis* from past studies

	*Arabidopsis* crown group	Arenosa/ Lyrata	LyrataNA/ LyrataEU	method	dataset	calibration
Beilstein et al. (2010) [58]	12.92 my	8.17 my	NA	BEAST	*ndh*F/*PHYA*	fossils
Hohmann et al. (2015) [3]	5.97 my	1.29 my	NA	BEAST	73 cp genes	fossils
Novikova et al. (2016) [25]	5.72 my	0.6 my	0.34 my	BEAST	complete cp genome	secondary, based on Hohmann et al. (2015) [3]
Koch et al. (2000) [59]	5.1–5.4 my	NA	NA	*K*s-simulation and permutation	*Chs* and *Adh*	synonymous substitution rate
Pyhäjärvi et al. (2012) [40]	NA	NA	69,000 generations (0.138 my assuming a 2-years generation time)	MIMAR	19 nuclear loci	mutation rate from Ossowski et al. (2010) [60]
Ross-Ibarra et al. (2008) [39]	NA	NA	19–32 ky	ms	77 nuclear loci	mutation rate from Koch et al. (2000) [59]

*LyrataNA* North American, *LyrataEU* European

### Directional gene flow and the origin of the hybrids

Directional gene flow between *Arabidopsis* species seems to be not unusual and has been described in hybrid *A. suecica* (maternal *A. thaliana* X paternal *A. arenosa*) and *A. kamchatica* (*A. lyrata* X *A. halleri*), both of hybrid origin, and introgressed *A. lyrata* (maternal *A. lyrata* X paternal *A. arenosa*) [7, 23, 24, 27, 32]. *Arabidopsis kamchatica* evolved several times independently (polytopic origins) and maternal contribution depends on the regions considered (e.g., *A. lyrata* as maternal parent in China, *A. halleri* as maternal parent in Japan). Combining these few examples there is some evidence that *A. lyrata* more often serves as maternal parent in introgression and hybridization scenarios, whereas *A. arenosa* plays a prominent role as paternal donor. Exactly this situation has been introduced in a suture zone along the Danube valley in the Wachau region in Lower Austria [7] and has been significantly demonstrated herein (Figs. 1, 4). Predictions from elevated mutational rates of plastid evolution in Lyrata when compared to Arenosa suggest that maternal Lyrata hybrids should have a lower fitness because of potential cytonuclear incompatibilities [33], but the opposite seems to be true in this natural introgression zone. Almost all tetraploid introgressed Lyrata have a plastid type belonging to Lyrata, indicating Arenosa usually acts as a pollen donor in the wild. This indicates that strong postzygotic and exogenous factors (e.g., climate and soil composition) act on first generation hybrids, thereby counteracting endogenously caused fitness constraints. One exception to this observation however could be in pop. One hundred thirty seven in the south of the introgression zone, where sample 137-c has a plastid type closely related to the neighboring pop. Two hundred one from Arenosa. The recent divergence suggests secondary contact of these taxa after the last glacial maximum in the Holocene. Although cytonuclear incompatibilities have been hypothesized to be the cause for the asymmetry of fitness in Arenosa and Lyrata crosses [33], maternal effects on gene expression might also play a role in the fitness asymmetry observed. In intraspecific *A. lyrata* hybrids, a strong bias towards the expression of maternal alleles was detected [61]. These effects could be mitigated by the high amount of introgression in the genome in pop. One hundred thirty seven (~57% of Arenosa, as indicated by STRUCTURE analysis), thus allowing for gene flow also in the reverse direction.

On a smaller scale gene flow from Lyrata to Arenosa can be observed in several populations, although putatively followed by backcrossing to Arenosa, not Lyrata, as indicated by plastid types. Results from STRUCTURE show that not only tetraploid Lyrata, but also the nearby populations of Arenosa show traces of introgression. This has not been shown before based on microsatellite loci used by Schmickl and Koch [7], and the extent observed in this study is somewhat surprisingly high. All populations of Arenosa from the study area investigated here show traces of Lyrata genome. While partial genetic assignment to the Lyrata cluster had been detected for populations directly adjacent to the introgression zone before, more specifically in the area around Mariazell in the eastern Austrian Forealps in the south and in the Wachau region at the north shore of the Danube river in the north [7], the results in this study indicates also partial assignment of an Arenosa population in the southern Czech Republic to Lyrata. Moreover, admixture was detected south of the introgression zone as far as to the distribution area of small range endemic and high alpine taxon *A. arenosa* subsp. *arenosa* var. *intermedia*. (pop. 210; Fig. 1). Tetraploid Carpathian Arenosa show no assignment to the Lyrata cluster at all, thus making polyploidy as the only explanation rather unlikely. Nevertheless, the traces of Lyrata genome found in high-alpine *A. arenosa* subsp. *arenosa* var. *intermedia* are surprising, considering the highly endemic and isolated distribution of this taxon in formerly alpine refugia.

Our findings with remarkable high amounts of Lyrata genome reported here from all populations of Arenosa within our total study area in Austria stand in sharp contrast to a recent study of Arenosa in Austria also based on whole genome sequence data, which found introgression from Lyrata into Arenosa only in one single serpentine adapted population [62]. Furthermore, analysis of shared polymorphism indicates a high amount of unique variants in tetraploid Lyrata (Fig. 2), and analyses of reduced datasets including only one population of this taxon show that only part of this variation is shared between introgressed populations. The number of private alleles in tetraploid Lyrata is surprisingly high, considering that they allegedly arose through hybridization of diploid *A. lyrata* subsp. *petraea* with *A. arenosa* subsp. *borbasii*, both of which are well represented in our analyses. A recent hybridization event should result in a low number of private alleles in hybrids, as they recruit all their genetic variation from the parental species. The high amount might also suggest that the hybridization occurred long ago in the “southern” montane refugia and diversity hotspot (Fig. 4), and therefore new variants could accumulate in the populations and afterwards migrated north towards the Danube river system. Alternatively, we might argue that the parental species were not fully recovered in our sampling, e.g. that populations now extinct served as parents or that the other species of *Arabidopsis* that is present in Austria, *A. halleri*, also played a role in the origin of tetraploid Lyrata. Considering the high levels of gene flow between all lineages of *Arabidopsis* [25], this does not seem unlikely. A comprehensive survey of Novikova et al. 2016 [25] did not show extensive gene flow between *A. lyrata* and *A. halleri*, but a preliminary population-based microsatellite study (Koch et al., unpublished) exemplified various *A. halleri* populations that are introgressed with *A. arenosa*, and we might assume that via this “bridge” *A. halleri* alleles might be able to enter even the genome of tetraploid *A. lyrata*.

### Introgression and its potential for adaptive evolution – A perspective

We have shown herein that there was a primary introgression from Arenosa into a maternal Lyrata genomic background in the northeastern Forealps around the maximum glaciation of the Riss glaciation (135 kya) with glaciers forcing populations of Arenosa and Lyrata into ‘melting suture zones’ at lower elevation. These regions are also of intermediate and/or mixed bedrock types and might have provided the environmental heterogeneity to enable introgressed populations to adapt to the new environment. These populations have served as genetic source for the subsequent colonization of lowland regions towards the Danube river (Wachau). This finding also correlates with the fact that tetraploid Lyrata populations in the Wachau are found on siliceous and intermediate bedrock types (Fig. 1c). Consequently one might have to consider adaptive evolution acting on substrate specific genes as shown earlier for bedrock type adaptation in North American *Arabidopsis lyrata* [63]. Evidence from the maternally inherited plastid genome suggests that gene flow was effectively unidirectional with Lyrata as the maternal parent. This was explained partially with cytonuclear incompatibilities [33]. However, it is also likely that we have to consider trade-offs in ecological traits in introgressed populations. Arenosa is a short-lived biennial and mostly single rosette-forming plant, which is adapted to produce many seeds with high immediate germination rates enabling the species to colonize rapidly disturbed habitats. In contrast, European Lyrata is a long-lived perennial with creeping stolons. Its flowering season is prolonged and seed production is limited with seeds of delayed germination. Consequently, introgressed and colonizing Lyrata populations might combine different parental traits, which are under respective selective regimes. However, the reason for the preference of Lyrata as maternal background in tetraploid populations is unclear, and maybe this is related to intrinsic processes such as stabilizing the polyploid genome and enabling regular meiosis [29–31]. We have set-up reciprocal crossing experiments with most of the populations and are analyzing respective ecological key traits in the offsprings, that can be discussed within a reliable genomic context and an evolutionary spatio-temporal scenario.

## Conclusions

Here we present a first detailed analysis of an *Arabidopsis* introgression zone in Austria based on genome-wide polymorphism data. The complex and highly dynamic system of past and ongoing gene flow between *A. lyrata* subsp. *petraea* and *A. arenosa* subsp. *borbasii* provides a great opportunity to study a number of evolutionary processes, such as adaptive introgression, asymmetrical fitness and periglacial isolation of populations in detail. The focus of our study lies on describing the population structure of the study system. Results from previous studies were significantly confirmed, and a surprisingly high amount of introgression, including from outside the originally described introgression zone, was detected. This adds to recent evidence suggesting that in *Arabidopsis* restrictions to gene flow are caused by geographic barriers rather than by genetic incompatibilities and past inter-lineage gene flow occurred frequently. Using chloroplast phylogenomics and corresponding divergence time estimations, our study strongly suggests a late Pleistocene origin of tetraploid introgressed Lyrata in the Eastern Austrian Forealps, most likely during the last inter- and periglacial period. The hybrids subsequently expanded northwards and colonized the entire introgression zone in the lowland Danube river system, with secondary contact to Arenosa possibly occurring after the LGM in the North close to the Bohemian Massif..

## Methods

### Plant material and sampling strategy

**Fig. 6:**
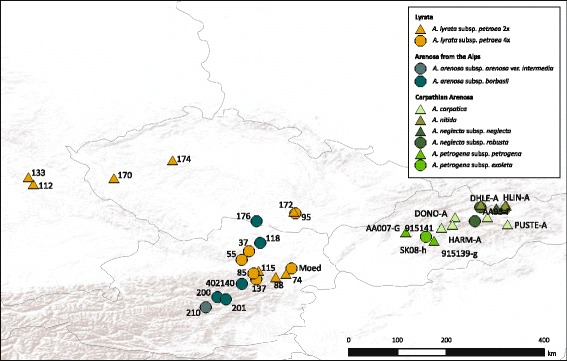
Sample locations of all populations included in this study. Taxa are indicated with color codes, diploids are shown with triangles and tetraploids with circles. The map was created under the Open Data licence Creative Commons 3.0 Österreich (CC BY 3.0 AT)

Focusing on an inter-species introgression zone in Eastern Austria stretching from the Northeastern Forealps to the Danube river we investigated 71 accessions in total representing samples of the Arenosa and Lyrata lineage [25]. Based on previous results [7, 27] transects were sampled representing clinal variation but also potential centers of genetic diversity and glacial refugia. The sampling also included individuals from Southeastern Germany, Czech Republic and Slovakia to set the study system into a broader phylogeographic and evolutionary context. The final dataset included diploid and tetraploid Lyrata, introgressed tetraploid Lyrata, various tetraploid Arenosa as putative parental source and diploid species from the Arenosa lineage representing the ancestral diploid Arenosa genepool. Sampling is displayed in Fig. 6 and further details are given with Additional file 2. Vouchers have been prepared and are deposited with the herbaria in Heidelberg (HEID) and Zurich (UZH). Details are provided with Additional file 2.

Phylogenetic analyses included *Arabidopsis cebennensis* as closest relative to Lyrata and Arenosa as outgroup taxon [28]. For plastome analyses sequences of *Arabidopsis thaliana* and *Capsella bursa-pastoris* were retrieved fromNCBI/Genbank and from a recent study focusing on the Brassicaceae family (Hohmann et al., 2015 [3]; *Capsella rubella* and *Camelina sativa*).

### DNA isolation

Total genomic DNA was extracted from herbarium vouchers, silica dried or fresh leaf material either following the CTAB protocol [64] or using the Invisorb Spin Plant Mini kit (STRATEC Biomedical AG, Birkenfeld, Germany). Fresh material was collected in the Botanical Garden Heidelberg and immediately frozen in liquid nitrogen until further use. Dried leaf material was immediately silica dried or taken from herbarium vouchers. Depending on leaf size, one to three leaves were used per extraction. Material was homogenized either by grinding with a pistil on liquid nitrogen, or using a Precellys®24 homogenizer (Bertin Technologies, Montigny-le-Brettonneux, France) at 5000 rounds per minute for two times 10 s with five 2.5 mm glass beads. The Invisorb Spin Plant Mini kit was used following the manufacturer’s instructions, including the optional step of RNA digestion. The following modifications were applied to the CTAB protocol: DNA pellets were washed twice with 70% ethanol and then dissolved in 100 μl TE-buffer (10 mM Tris-HCl, 1 mM EDTA, pH 7.5) supplemented with 2 units of RNase A, RNA digestion was performed at 37 °C for 1 h. Quality (high molecular weight) and quantity (> 5 ng/μl) of DNA was checked rigorously prior to library preparation. DNA quality and fragment length was checked on 1% agarose gels, and concentration was assessed via fluorescence spectroscopy using a high-sensitivity, double-stranded DNA specific dye with the Qubit® dsDNA HS Assay (Thermo Fisher Scientific, Waltham, Massachusetts, USA).

### Library preparation and next-generation sequencing

Libraries were sequenced at the Deep Sequencing Core Facility, University of Heidelberg, and were prepared from total genomic DNA with the Illumina TruSeq Kit (Illumina, Inc., San Diego, California, U.S.) in paired-end mode with insert size 200–400 base pairs (bp). Read length was 100 bp. Samples were barcoded for multiplexing, and on average 4 tetraploid or 8 diploid individuals were sequenced on one lane to obtain intermediate sequencing depth. Sequencing was conducted on Illumina HiSeq2000 (Illumina, Inc.).

For details regarding library preparation method, see Additional file 2.

### Assembly and annotation of plastid genomes

Assembly of plastid genomes generally followed the procedure described in Hohmann et al. (2015) [3] and Novikova et al. (2016) [25]. To recover the complete plastome sequence the reads were assembled de novo in CLC Genomics workbench version 6.0.4 (CLC bio, Aarhus, Denmark). Failed reads were discarded upon import, and imported reads were trimmed for sequencing quality as well as for remaining adapter sequences. A minimum per-base quality of 0.001 (corresponding to a phred-based score of 30) was applied, with a minimum remaining length of 50 bp. Only paired reads were used further.

The legacy version of the CLC Genomics de novo algorithm was used with distance settings matching the insert size of sequencing libraries. Sequences were required to have a sequence similarity of 0.9 over a length fraction of 0.9 for assembly, and the default parameters were used for mismatch, insertion and deletion cost (2, 3, and 3, respectively). Random assignment was used for non-specific matches, and conflicts were resolved by voting. An automatic word size was applied, and minimum contig length was generally 2000 bp, but adjusted depending on read number to ensure sufficient computational resources. Reads were mapped back to contigs to achieve an accurate assembly and be able to detect inconsistencies. Contigs belonging to the plastid genome are easily identified by their high coverage, as plastids have a high copy number in each cell, comparable to that of nuclear encoded ribosomal DNA and approximately 10 times higher than that of the mitochondrial genome [65]. The browser version of NCBIs nucleotide blast was used to confirm the identification as plastid sequence with default settings and detect the sequence direction. These were manually aligned to a reference in PhyDE version 0.9971 [66]. Already assembled sequences were used as a reference, and since here only additional individuals from already known populations were sequenced, the already assembled genomes from respective populations were used. Contigs often overlapped, but where they did not the sequence of the reference was inserted into a pseudo genome. This in turn was used as a reference for mapping in a round of quality control. The legacy version of the CLC Genomics mapping algorithm was used with the same settings as for de novo assembly to map all reads back to the hybrid sequence of reference and newly assembled contigs. Subsequently, Variant Detection was conducted in CLC Genomics to identify differences between reads and reference, ignoring non-specific matches and broken pairs. To find all differences such as SNPs and short indels, the minimum coverage was set to 1 and variant probability to 0.1. As the plastid genome is haploid, the maximum expected number of variants was 1. Long indels, which will not be identified by Variant Detection, were located manually. If variants were found, the newly assembled genome sequence was adjusted and the mapping as well as variant detection steps were repeated until no further differences could be detected.

Sequences were annotated in Geneious version 7.1.7 (Biomatters Ltd., Auckland, New Zealand). This was achieved by aligning the newly generated plastid genome sequences to the *A. thaliana* reference (Genbank accession AP000423) using the automatic alignment parameters, and then transferring the annotations. Annotations were checked manually for the correct assignment of start- and stop codons, and annotation features were adjusted if needed.

### Alignments of plastomes

Complete plastid genomes were aligned using the MAFFT version 7.017 [67–69] plugin in Geneious v7.1.7 (Biomatters Ltd., Auckland, New Zealand) with the FFT-NS-ix1000 algorithm. The default parameters 200 PAM/k = 2 scoring matrix, gap open penalty of 1.53 and offset value of 0.123 were used. *C. rubella*, *C. bursa-pastoris* and *C. sativa* were included as outgroup to the genus *Arabidopsis*, and *A. thaliana* as well as *A. cebennensis* were included as relatives to the Arenosa and Lyrata. Subsequently, the alignment was cut into segments representing single exons, introns and intergenic spacers. Regions with two overlapping annotations were used only once. The segments on the Inverted Repeat were included only once, resulting in a dataset of 265 alignments. All alignments were realigned using the stand-alone version of MAFFT version 7.123b [69], with the iterative refinement method with local pairwise alignment information (command line arguments: --localpair --retree 6 --maxiterate 16), and gapped positions were excluded using Gblocks version 0.91b [70] with default block settings, except for minimum block length, which was set to 2 bp. Seven alignments were excluded because of their block length. These were generally the short sequences sharing two annotations. The remaining 257 alignments were used for further analysis.

PartitionFinder version 1.1.1 [71, 72] was used to find an optimal partitioning scheme and evolutionary model for the alignments. Only models implemented in BEAST were tested, with branch lengths unlinked and BIC for model selection in a greedy search. The best partitioning scheme consisted of 2 subsets. One subset contained mostly coding regions as well as all segments from the Inverted Repeat region, with best model GTR + Γ, possibly caused by the slower substitution rate of the Inverted Repeat region [73] and coding regions. The second subset contained mostly intergenic regions and introns, with best evolutionary model GTR + Γ + I. For details on partitioning scheme see Additional file 3. Alignments are available with Additional file 4.

### Phylogenetic reconstructions

Maximum likelihood (ML) trees of plastome sequence data were reconstructed using RAxML version 8.1.16 [74]. The partitioned dataset was used as input, with individual per partition branch length optimization, for a rapid bootstrap analysis with 1000 bootstrap replicates followed by a thorough ML search. RAxML does not allow for individual per partition evolutionary models, therefore GTRGAMMA was used for both partitions, also following the recommendation of the RAxML user manual. The clade of *C. sativa*, *C. rubella* and *C. bursa-pastoris* was set as outgroup. Output was visualized in FigTree version 1.4.1 [51].

### Divergence time estimations

Divergence times of plastome lineages were estimated using BEAST version 1.7.5 [51]. The partitioned alignments according to the best partitioning scheme as described above were used, with unlinked substitution and clock models and a combined tree. GTR + G and GTR + G + I were used as substitution models for the two subsets, respectively, with 4 gamma categories. The clock model was uncorrelated log normal relaxed clock [75] with starting rate 1.0 × 10^−4^ for both subsets, and estimated rates. Tree prior for the combined tree was Speciation: Birth-Death Incomplete Sampling [76]. A user-defined starting tree was generated by converting the ML tree into a chronogram and adjusting node ages to fit the priors in R version 3.2.3 [77] using the function ‘chronos’ included in the R package APE version 2.13 [78].

Secondary calibration was applied following Novikova et al. (2016) [25] and using the estimates from Hohmann et al. (2015) [3]. The split between *A. thaliana* and the rest of *Arabidopsis*, i.e. the *Arabidopsis* crown age, was set to 5.96 mya, and the split of the two outgroup genera, *Camelina* and *Capsella*, was set to 7.35 mya [3]. A normal distribution with standard deviation 1.0 was used for both of these calibration points, as this was a good fit with the 95% HPD intervals from Hohmann et al. 2015 [3]. The tree root height, i.e. the *Arabidopsis* stem age, was constrained using a normal distribution with a mean value of 8.16 mya and standard deviation of 1.0.

Two independent MCMC chains were run for 1 × 10^8^ generations, sampling parameters every 1 × 10^4^ generations. Subsequently, the two analyses were combined in LogCombiner version 1.7.5 [51], discarding the first 1 × 10^7^ generations of each run as burn-in, and the resulting 18,000 trees were combined to a maximum clade credibility tree with median node heights in TreeAnnotator version 1.7.5 [51]. The tree including the 95% HPD intervals of divergence time estimates was visualized in FigTree version 1.4.1 [51].

The combined tree file including 95% HPD intervals for all nodes are available in Additional file 5.

### Mapping of the reads form the nuclear genome

Reads of Arenosa and Lyrata samples were mapped against the *A. thaliana* reference genome (TAIR: https://www.arabidopsis.org). The also available genome sequence of *A. lyrata* was not used in order to prevent a bias of better mapping of one given species to an ingroup reference. Mapping was performed using the BWA-MEM [79] algorithm from BWA version 0.7.8 [80] with an increased penalty for unpaired read pairs to 15 and default settings for all other parameters. Duplicate mapped reads were then removed with SAMtools version 0.1.19 [81, 82]. Indel realignment was performed with IndelRealigner from GATK version 3.3 [83], and the alignment was filtered for primary and unique reads with SAMtools (flags –F 256 –q 5). Since the sampling included diploid as well as tetraploid individuals, variant calling could not be performed for all samples simultaneously. Therefore, variants were first called for individual samples in GATKs Unified Genotyper for both SNPs and indels for all confident sites [84], and subsequently combined using GATKs CombineVariants with filtered sites treated as uncalled. In addition to quality filters applied by default by GATK we used only sites of intermediate coverage, thereby excluding potential duplicated regions and pseudo-genes. Coverage distribution was calculated with GATKs Pileup, and coverage density was calculated. GATKs CallableLoci tool was used to find intervals with a minimum coverage corresponding to the ploidy level of the sample and a maximum coverage of 0.95 of the coverage density, and variant calling was performed only in intervals that fulfilled the criteria for coverage.

After combining the variants, a set of 15,454 genes was extracted with the intersect function of the BEDTools program collection [85]. These genes were already used in Novikova et al. (2016) [25] as they were well covered in a data set of genomic sequence from all taxa within the genus *Arabidopsis,* and they were proven to be single-copy using the same coverage criteria as described above. This resulted in a subsampling of the complete genome comprising 39,046,724 bp including 3′- and 5′-UTR as well as introns, of which 7,581,148 had SNPs in the dataset. Only SNPs were used for downstream analyses, and only variant sites were selected. An additional filtering step for sites with 50% missing data or more, or with frequency 1 for the derived allele, i.e. invariant sites, resulted in 5,473,967 sites, out of these 1,414,525 are synonymous SNPs. Annotations were added using SnpEff version 4.2 [86].

### Genetic clustering analysis

Clustering analysis based on nuclear genome data was conducted only on a subset of data. This was necessary to rescale to a computationally feasible dataset, and was done by randomly choosing a fraction of 0.05 of synonymous variants from the original file using GATKs SelectVariants. Ten independent datasets were generated, and used as input for STRUCTURE analyses. Since the function ‘fraction’ uses a probabilistic approach, the number of SNPs varies between input files (between 70,383 and 70,475). To include diploid and tetraploid individuals into the same analysis, diploid data was duplicated similar to the procedure described in Hohmann et al. 2014 [28].

STRUCTURE version 2.3.4 [45, 87] was run with allele frequencies correlated in the admixture algorithm. Ten thousand MCMC steps were discarded as burn-in, and followed by 20,000 iterations for K = 1–6 (number of clusters). Instead of 10 independent runs for each K, as is commonly done when using STRUCTURE, the 10 subsets of data were used as input for different runs.

To demonstrate that the genetic assignment of individuals is robust to the random subsampling performed here, CLUMPP version 1.1.2 [88] was used to find the matching permutation for each run. Input files for CLUMPP were generated with the stand-alone version of structureHarvester version 0.6.94 [89]. The R script structure-sum [90] was used to calculate similarity between runs as well as determine the best number of clusters according to the method described by Evanno et al. (2005) [46]. Results from structure-sum are shown in Additional file 6. Distruct version 1.1 [91] was used to plot mean STRUCTURE results for the optimal number of clusters.

### Shared polymorphism among taxa and cytotypes

To determine the number of shared polymorphisms within the nuclear data, four groups were compared, namely diploids and tetraploids of Lyrata and Arenosa, respectively. Tetraploid Lyrata from outside the introgression zone (pops. 95 and Moed) were not considered in this analysis. In addition, the same analyses were conducted using subsets of tetraploid Lyrata comprising only samples of one of the four populations from the introgression zone. Only biallelic SNPs were selected, and sites with missing data were discarded. The amount of alleles shared between groups was counted and Venn diagrams were drawn in R version 3.2.3 [76] using the library VennDiagram version 1.6.17 [92], and further details are shown with Addiitonal 7.

### SplitsTree analysis of nuclear data

The full set of variants in the 15,454 genes, over all samples, with filtering for more than 50% missing data as described above, i.e. 5,473,967 SNPs, was used for network reconstruction. Here also multiallelic SNPs could be used. Fasta files were generated in GATK using FastaAlternateReferenceMaker [82]. IUPAC ambiguity codes were inserted for heterozygous SNPs. A phylogenetic network was reconstructed in SplitsTree version 4.14.2 [47] using the equal angle NeighborNet with 1000 bootstrap replicates.

## Additional files

Additional file 1: Result of SplitsTree network reconstruction. (PDF 770 kb)

Additional file 2: Accession list and population details. (XLSX 28 kb)

Additional file 3: Partitioning scheme of plastid DNA sequence data for phylogenetic analysis. (TXT 14 kb)

Additional file 4: Alignment of plastid DNA sequence data for phylogenetic analysis. (TXT 9059 kb)

Additional file 5: Combined tree file (plastome data) with BEAST output. (TXT 147 kb)

Additional file 6: Structure-sum output, analysis of deltaK and similarity values between STRUCTURE runs. (PDF 165 kb)

Additional file 7: VENN diagrams with absolute numbers of SNPs. (PDF 2549 kb)

## Abbreviations

Kya: Thousand years ago
ML: Maximum likelihood
Mya: Million years ago
