# Correction to: An *Arabidopsis* introgression zone studied at high spatio-temporal resolution: interglacial and multiple genetic contact exemplified using whole nuclear and plastid genomes

**DOI:** 10.1186/s12864-018-4614-0

**Published:** 2018-04-11

**Authors:** Nora Hohmann, Marcus A. Koch

**Affiliations:** 1 Center for Organismal Studies (COS) Heidelberg/Botanic Garden and Herbarium Heidelberg (HEID), University of Heidelberg, Im Neuenheimer Feld 345, D-69120 Heidelberg, Germany; 2 Present address: Department of Hohmann and Koch BMC Genomics (2017) 18:810 Page 16 of 18 Environmental Sciences, Botany, University of Basel, Hebelstrasse 1, CH-4056 Basel, Switzerland

## Correction

Upon publication of the original article [1], the authors had flagged that there was an error in Fig. 1c, as the key in this figure was displaying incorrectly. The colours had not displayed in the key in the final published article, and instead appear as plain white.

An updated version of Fig. 1 is included with this article to ensure the original figure can be interpreted (Corrected Fig. 1).

The publisher apologises for this error.

Corrected Fig. 1.

**Fig. 1:**
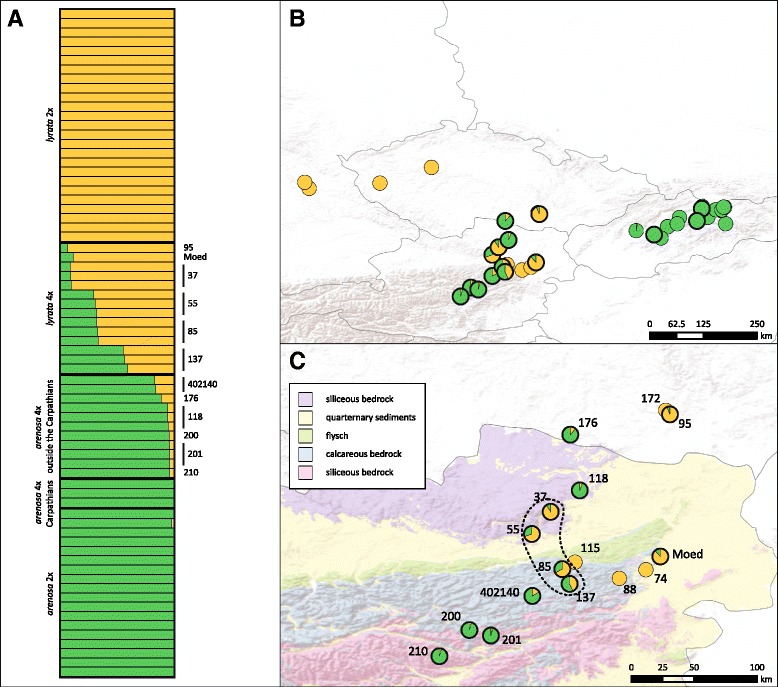
Genetic clustering analysis from STRUCTURE [45]. Results for mean values over 10 independent runs at K = 2 are shown. Runs are based on 10 randomly sampled subsets of the complete dataset of 15,454 genes, using a fraction of 0.05 of synonymous sites per subset. **a** bar plot showing the genetic assignment of each sample, sorted by taxon and population. **b** Mean values over all individuals as pie charts in each population in the complete sampling area. **c** Mean values over all individuals in each population in enlarged suture zone in northeastern Austria and adjacent regions. The Wachau region is indicated with a dashed line. Tetraploid populations are marked by black circles around the pie charts. Bedrock type was categorized based on the geological map of Austria (KM500 Austria from http://www.geologie.ac.at) under the Open Data licence Creative Commons 3.0 Österreich (CC BY 3.0 AT)

## References

[1] Hohmann N, Koch MA (2017). An Arabidopsis introgression zone studied at high spatio-temporal resolution: interglacial and multiple genetic contact exemplified using whole nuclear and plastid genomes. BMC Genomics.

