# Supplementary material for: An Arabidopsis introgression zone studied at high spatio-temporal resolution: interglacial and multiple genetic contact exemplified using whole nuclear and plastid genomes
Source: BMC Genomics. 2017 Oct 23;18:810. doi: 10.1186/s12864-017-4220-6 (PMC5651623; doi:10.1186/s12864-017-4220-6)
Supplement: Supplementary file 1 — Result of SplitsTree network reconstruction. (PDF 770 kb) [file 12864_2017_4220_MOESM1_ESM.pdf]

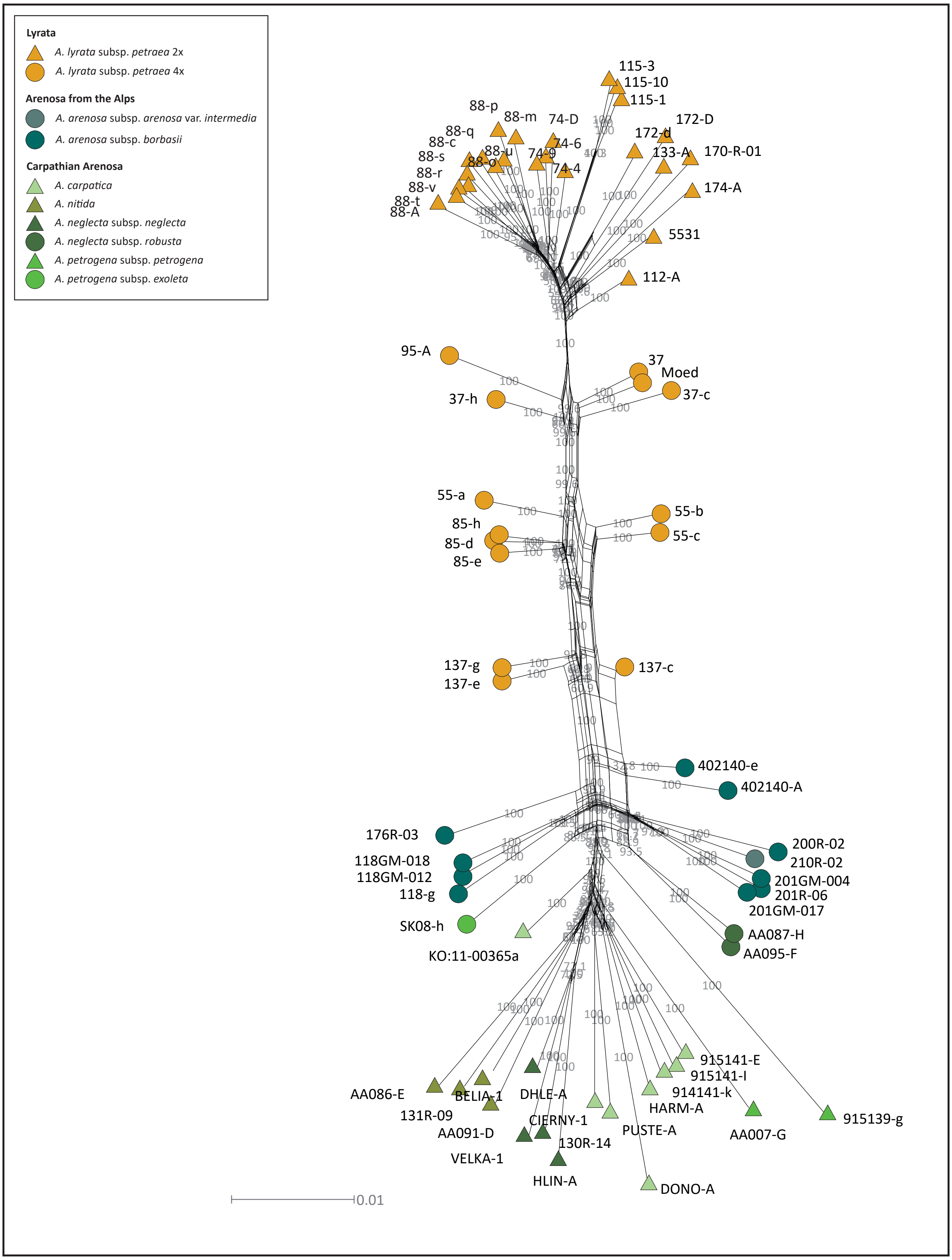

Additional File 1. SplitsTree [47] equal angle NeighborNet based on 5,473,967 SNPs from the nuclear genome. Bootstrap values from 1000 replicates are indicated in grey. Taxa are indicated with color codes, diploids are shown with triangles and tetraploids with circles.
