# Supplementary material for: An Arabidopsis introgression zone studied at high spatio-temporal resolution: interglacial and multiple genetic contact exemplified using whole nuclear and plastid genomes
Source: BMC Genomics. 2017 Oct 23;18:810. doi: 10.1186/s12864-017-4220-6 (PMC5651623; doi:10.1186/s12864-017-4220-6)
Supplement: Supplementary file 6 — Structure-sum output, analysis of deltaK and similarity values between STRUCTURE runs. (PDF 165 kb) [file 12864_2017_4220_MOESM6_ESM.pdf]

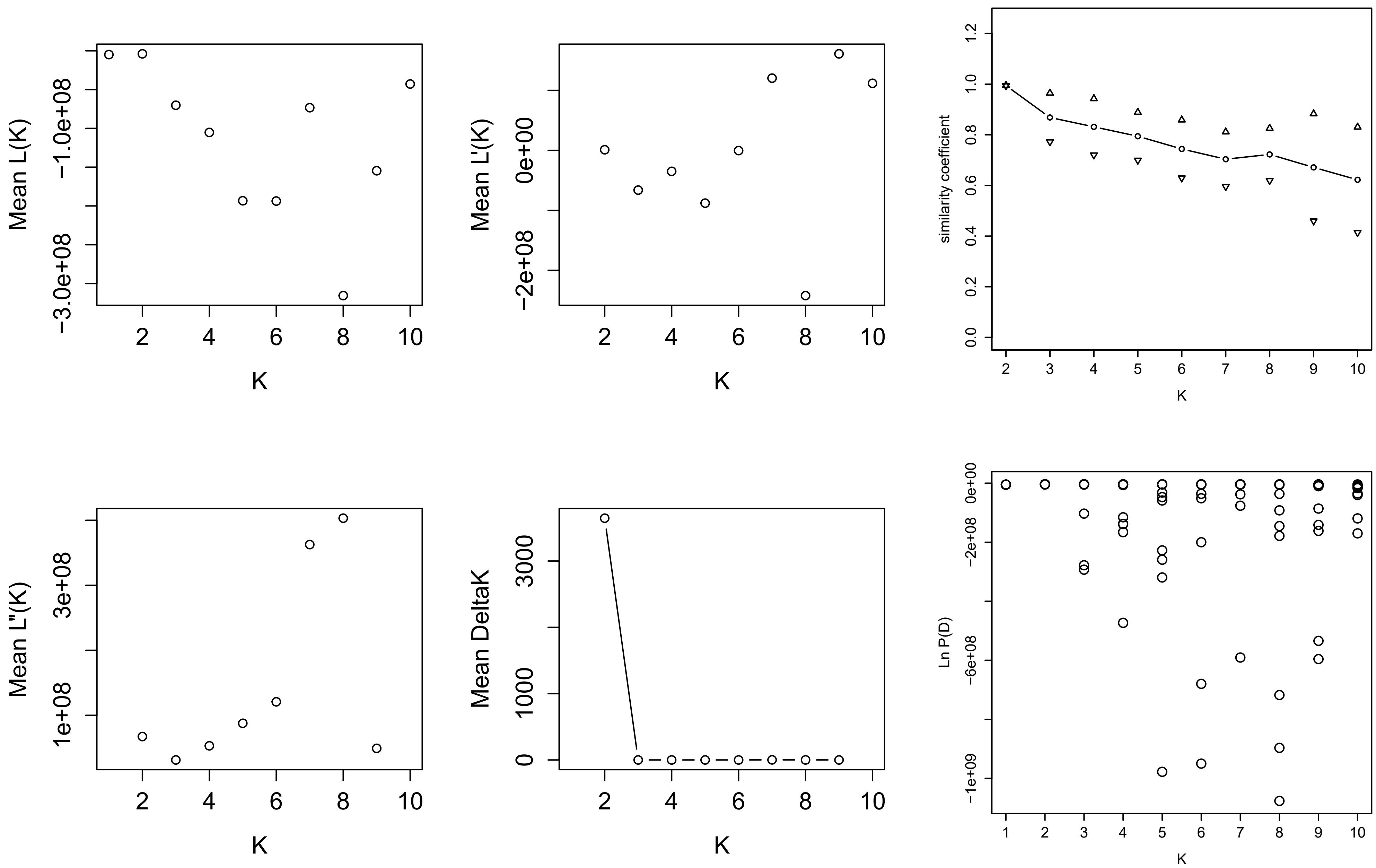

Additional File 6. Structure-sum [85] output, analysis of deltaK and similarity values between 10 independent STRUCTURE [45,82] runs.
