# Supplementary figures and images for: An Arabidopsis introgression zone studied at high spatio-temporal resolution: interglacial and multiple genetic contact exemplified using whole nuclear and plastid genomes
Source: BMC Genomics. 2017 Oct 23;18:810. doi: 10.1186/s12864-017-4220-6 (PMC5651623; doi:10.1186/s12864-017-4220-6)

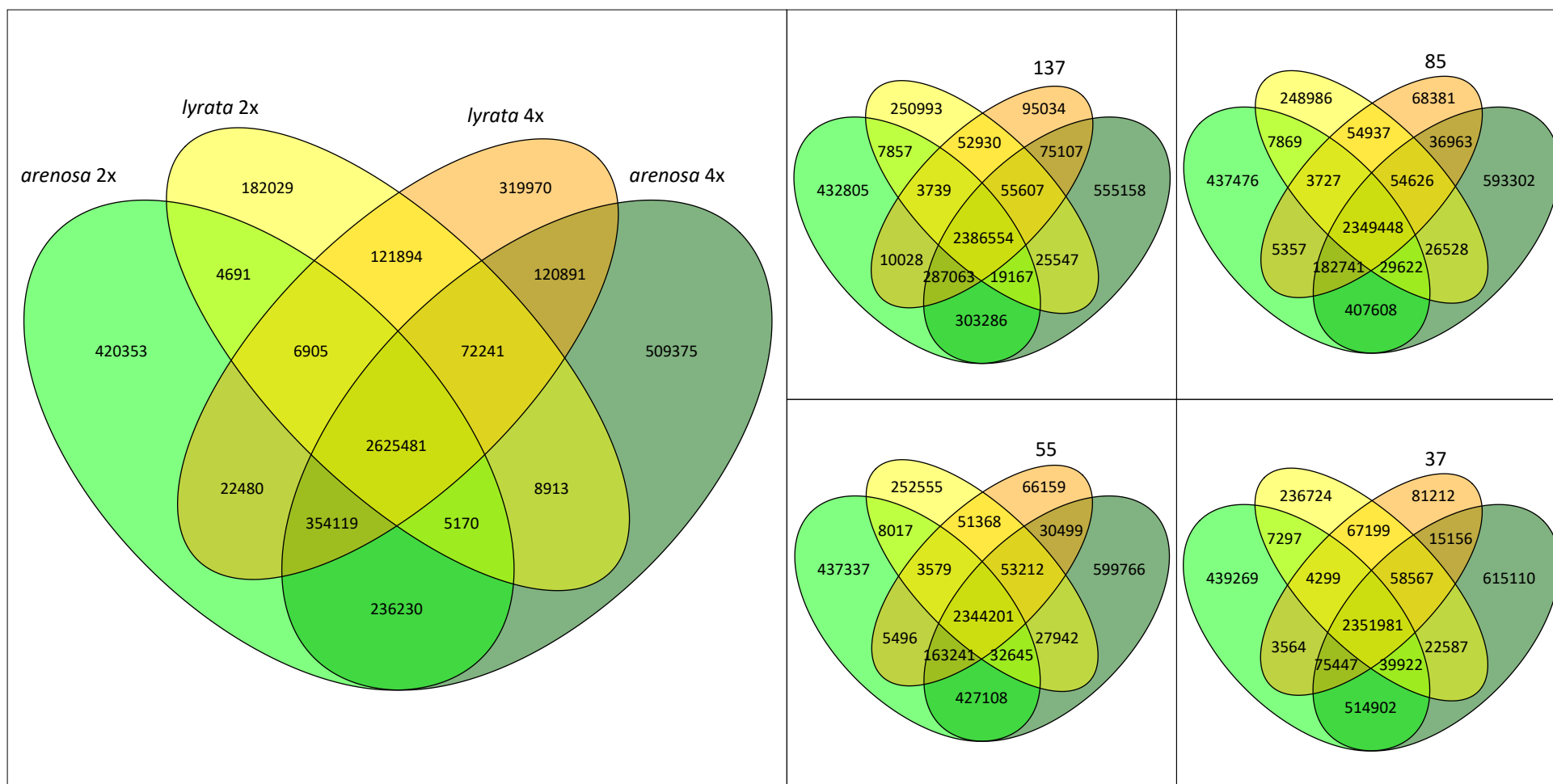

Additional File 7.

Supplement: Supplementary file 7 — VENN diagrams with absolute numbers of SNPs. (PDF 2549 kb) [file 12864_2017_4220_MOESM7_ESM.pdf]
